# GPR35 Mediates the Proliferation and Adipogenesis of Goat Intramuscular Preadipocytes Through the cAMP/PKA Signaling Pathway

**DOI:** 10.3390/ani16162560

**Published:** 2026-08-17

**Authors:** Fei Wang, Qiuchi Meng, Jingwen Gao, Tingyao Cui, Ziyan Qi, Nan Zhao, Yaqiu Lin, Jiani Xing, Youli Wang, Yanyan Li

**Affiliations:** 1 Key Laboratory of Qinghai-Tibetan Plateau Animal Genetic Resource Reservation and Utilization, Ministry of Education, Southwest Minzu University, Chengdu 610041, China; swunwangf@163.com (F.W.); m1416779568@163.com (Q.M.); xiaozhao202208@163.com (N.Z.); linyq1999@163.com (Y.L.); 80300244@swun.edu.cn (J.X.); wangylwy@163.com (Y.W.); 2 College of Animal & Veternary Sciences, Southwest Minzu University, Chengdu 610041, China; 18313606778@163.com (J.G.); 18100477995@163.com (T.C.); 18712193096@163.com (Z.Q.); 3 College of Modern Agriculture, Sichuan Sanhe College of Professionals, Luzhou 646200, China

**Keywords:** goat, GPR35, adipogenesis, proliferation, cAMP/PKA signaling pathway, intramuscular preadipocytes

## Abstract

GPR35 is a member of the G-protein-coupled receptor family and existing studies have shown that its expression may be negatively correlated with fat deposition in mice. However, the exact role of GPR35 in goat intramuscular preadipocytes’ adipogenic differentiation remains unknown. This study is the first to demonstrate that GPR35 inhibits the maturation and differentiation of goat intramuscular preadipocytes via activating the cAMP/PKA signaling pathway under in vitro culture conditions. The functional experimental results showed that GPR35 overexpression suppressed the adipogenic differentiation, lipid synthesis, and proliferation capacity of goat intramuscular preadipocytes, and concurrently downregulated the expression of adipogenic differentiation marker genes and cell proliferation marker genes. Conversely, GPR35 knockdown promoted lipid accumulation, reduced intracellular cAMP levels, and ultimately enhanced the differentiation and proliferation capacity of intramuscular preadipocytes. Treatment with the cAMP inhibitor SQ22536 and PKA phosphorylation inhibitor H89 successfully reversed the phenotypic changes induced by GPR35 overexpression, including abnormal lipid droplet morphology, decreased triglyceride (TG) content, and inhibition of the expression of genes related to adipocyte differentiation/proliferation/metabolism (such as CDK2, PCNA, and HSL). These results confirm that GPR35 mainly regulates intramuscular fat deposition in goats through the cAMP/PKA pathway, providing a new target for elucidating the molecular mechanism of lipid metabolism and goat molecular breeding.

## Simple Summary

Intramuscular fat, the fine “marbling” distributed in goat muscle, is the core determinant that directly shapes the tenderness, juiciness, flavor and overall palatability of goat meat. In this goat-focused study, we identified that the protein GPR35 functions as a critical regulatory switch for intramuscular adipocytes. It acts through the well-characterized cAMP/PKA signaling cascade inside cells to promote the proliferation of preadipocytes resident in goat skeletal muscle and facilitate the terminal differentiation of these immature cells into mature lipid-laden adipocytes. This finding will support the development of more scientific breeding and feeding strategies, to produce high-quality goat meat with appropriate intramuscular fat content and superior eating quality.

## 1. Introduction

Goats represent an ideal ruminant livestock model for investigating climate change adaptation and the sustainability of food security. Alongside rising public health awareness and continuous upgrading of dietary preferences, consumer demand for high-quality goat meat has maintained steady growth [1,2,3]. Intramuscular fat (IMF) refers to the fat deposited within muscle fibers, which has an important impact on the sensory qualities of meat products, such as flavor, tenderness, juiciness, and color. Its content and distribution are one of the important indicators for evaluating the quality of goat meat [4,5]. The intramuscular adipogenesis in livestock is a sophisticated multi-stage biological mechanism that critically encompasses three core sequential events: clonal expansion of intramuscular preadipocytes, temporal differentiation of intramuscular preadipocytes, and progressive lipid droplet accumulation [6,7]. Elucidating these regulatory mechanisms in goat models will lay a critical theoretical foundation for goat meat quality improvement and the high-quality development of the goat industry.

G protein-coupled receptors (GPCRs) are the largest and most diverse superfamily of transmembrane proteins in mammals [8]. Their core feature is a conserved seven-transmembrane (7TM) domain [9,10]. By binding to agonists (such as hormones and neurotransmitters), they trigger conformational changes, which in turn activate heterotrimeric G proteins (α/β/γ subunits) to regulate downstream effectors and initiate various cellular responses [11,12]. Therefore, they are widely involved in physiological processes such as neural conduction, immune regulation and metabolic regulation, and are important targets for the development of drugs for chronic diseases such as obesity and diabetes [13,14,15,16,17]. In addition, GPCRs also participate in the regulation of processes such as adipocyte differentiation, lipid synthesis and breakdown through interactions with lipid ligands, affecting the body’s energy metabolic balance [14,18,19]. GPR35 is a member of the rhodopsin-like class A G protein-coupled receptor (GPCR) family [11]. It can regulate signaling pathways such as cAMP, Rho, and PLC by coupling with multiple G proteins (Gα_13_, Gαi/o) and recruiting β-arrestin-2 [20,21]. Moreover, it activates oncogenic signals independently of G-proteins (via interaction with the sodium–potassium pump) and contributes to the onset and progression of inflammation, metabolic disorders, and neoplasms [20,22]. Cumulative evidence demonstrates that GPR35 expression is significantly associated with conditions resulting from metabolic dysfunction, including obesity and diabetes [21,23]. As a sensor for exercise-induced kynurenic acid (KYNA), GPR35 stimulates downstream signaling cascades, facilitating white adipose tissue browning and enhancing energy expenditure while suppressing adipose tissue inflammation, thereby improving whole-body energy homeostasis and metabolic health [24,25]. Using a murine hepatocyte model, Wei et al. performed lipidomics analysis and revealed that GPR35 activation enhances cholesterol esterification and expedites bile acid bioconversion via upregulating STARD4 expression [26]. Concurrently, it blocks the SREBP-1c-mediated lipogenic pathway, thereby markedly reducing hepatic lipid deposition [27]. Collectively, current research has established that GPR35 plays a conserved regulatory function in mammalian lipid metabolism; however, there has been no functional investigation focused on this gene in goats, a significant domestic ruminant species, especially regarding intramuscular lipid deposition.

This study utilized intramuscular preadipocytes isolated from Jianzhou Daer goats as the in vitro experimental model to elucidate the functional role of GPR35 and its underlying regulatory mechanisms in the proliferation and adipogenic differentiation of this cell type, providing novel molecular targets for basic research on goat meat quality improvement.

## 2. Materials and Methods

### 2.1. Test Animals

Goat intramuscular preadipocytes were isolated from the longissimus dorsi muscle of 3 healthy 7-day-old male Jianzhou Daer goats (Sichuan Tiandi Yang Biological Engineering Co., Ltd., Chengdu, China) under sterile conditions. The isolation and identification methods for preadipocytes were performed as previously described by Xu Q [28]. The primary intramuscular preadipocytes isolated from each individual healthy 7-day-old male Jianzhou Daer goat were defined as one independent biological replicate. Cells derived from each individual goat were cultured separately for subsequent experiments. For each biological replicate, cell suspension from the same batch was simultaneously seeded into 3 parallel wells as technical replicates. This study was approved by the Institutional Animal Care and Use Committee of Southwest Minzu University (Chengdu, Sichuan, China), and all animal procedures conformed to ethical treatment protocols.

### 2.2. Cultivation and Induced Differentiation of Goat Intramuscular Precursor Adipocytes

Cryopreserved goat intramuscular preadipocytes extracted from liquid nitrogen were promptly revived in a 37 °C water bath. Subsequent to low-velocity centrifugation, the cellular pellet was reconstituted in complete medium (DMEM/F12, 20% FBS, 1% Penicillin-Streptomycin Solution) for seeding, then incubated in a CO_2_ incubator (37 °C, 5% CO_2_). Upon achieving 80% confluence, with the cells fully covering the culture dish’s surface, subculturing or experimental plating was conducted. F3-generation cells were inoculated onto cell culture plates, and transfection of the cells was performed the subsequent day. At 12 h post-transfection, the culture medium was replaced with either complete medium or adipogenic induction medium (complete medium supplemented with 50 μmol/L oleic acid (Sigma, Tokyo, Japan)). At 48 h post medium change, cells were harvested according to the designated experimental protocol for subsequent downstream analyses.

### 2.3. Vector Construction, Chemical Synthesis of siRNA, and Cell Transfection

The overexpression vector of the GPR35 gene was obtained through previous construction in our laboratory. The CDS region of the GPR35 gene was ligated into the pEGFP-N1 vector linearized by restriction endonucleases, named OE. pEGFP-N1 plasmid was selected as the negative control (NC). The si-GPR35 directed against GPR35 was purchased from Gene Pharma (Shanghai, China). The sequences of si-GPR35 and the si negative control (si-NC) were as follows:

si-GPR35:

forward strand: 5′-AAGGGAGUCUUGCUCACCUTT−3′.

reverse strand: 5′-AGGUGAGCAAGACUCCCUUTT−3′.

si-NC:

forward strand: 5′-CAAUCGCCUUUGCUGUCAATT−3′.

reverse strand: 5′-ACGUGACACGUUCGGAGAATT−3′.

All transfection assays were conducted using TurboFect Transfection Reagent (Thermo, Waltham, MA, USA) according to the manufacturer’s protocol. Taking a 6-well plate as an example, the transfection system for overexpression is as follows: 2 μg of plasmid, 6 μL of transfection reagent, and 400 μL of DMEM/F12 medium. Similarly, the transfection system for interference is: 6 μL (20 μM) of siRNA (si-NC or si-GPR35), 6 μL of transfection reagent, and 400 μL of DMEM/F12 medium. After incubating at room temperature for 15 min, add the mixture to a 6-well plate. Replace the transfection medium with oleic acid induction medium after 12 h of transfection. Collect the cells after 48 h of culture.

### 2.4. Oil Red O and BODIPY Staining

F3-generation goat intramuscular preadipocytes were cultured in 48-well plates. Subsequent to cell transfection, the cells underwent induction for adipogenic differentiation and were subsequently harvested 48 h later. The 48-well plate was taken out, the supernatant was eliminated, and 1 mL of pre-chilled PBS was introduced to rinse the cells in each well. After a 15 min exposure to 4% formaldehyde, they were subsequently washed with PBS 2–3 times. Staining was conducted at ambient temperature utilizing an Oil Red O working solution for a duration of 30 min. Washing with PBS after staining facilitated microscopic examination and imaging. For semi-quantitative assessment of Oil Red O staining outcomes, 1 mL of 100% isopropanol was introduced to each well to remove the pigment. Subsequently, 200 μL of the extract was allocated to a 96-well plate, and the absorbance (OD value) was assessed at 490 nm utilizing a microplate reader. In a similar manner, following the fixation of the cells with 4% formaldehyde for 15 min, they were subsequently stained with BODIPY working solution and Hoechst for 30 min at ambient temperature. The cells were examined and captured under the fluorescent microscope (Olympus, Tokyo, Japan). Fluorescent images of BODIPY staining were acquired using an inverted fluorescence microscope, and 5 randomly selected fields per sample were subjected to quantitative analysis in a blinded manner. The relative fluorescence area was assessed using ImageJ 1.54g (National Institutes of Health, Bethesda, MD, USA), with the system default threshold applied for signal identification during quantification.

### 2.5. Triglyceride Assay

F3-generation intramuscular preadipocytes were inoculated into 6-well plates. Subsequent to cell culture, transfection, and 48 h of adipogenic differentiation, the culture medium was removed, and the cells were washed three times with pre-chilled PBS. The intracellular triglyceride (TAG) levels were assessed utilizing a Tissue Tri-glyceride Assay Kit (Applygen, Beijing, China, No. E1013). Test specimens were acquired by disrupting cells on ice using 200 μL of cell lysis solution for a duration of 10 min. The test specimens were combined with the working solution at a volume ratio of 1:19 in a 96-well plate while being shielded from light, and the absorbance (OD value) was recorded at 550 nm. To standardize the triglyceride levels among various groups, the protein concentration of the lysed samples was quantified utilizing a BCA protein assay kit (Boster, Wuhan, China).

### 2.6. MTT Analysis

F3-generation goat intramuscular preadipocytes were inoculated into 96-well plates. The cell plates were collected at 0, 24, 48, and 72 h following transfection for further examination. A total of 10 μL of MTT working solution (5 mg/mL, Solarbio, Beijing, China) was introduced into each well, then incubating in a CO_2_ incubator (37 °C, 5% CO_2_) for 4 h. Subsequently, 120 μL of DMSO solution was introduced into each well, and the plate was incubated for 15 min in a light-protected environment. Ultimately, 100 μL of the experimental sample was allocated to a 96-well plate, and the absorbance was assessed at a wavelength of 490 nm. Five technical repetitions were established for each specimen.

### 2.7. EDU Staining

F3-generation intramuscular preadipocytes were cultured in 48-well plates. Following transfection and a 48 h culture period, EdU staining was conducted utilizing the BeyoClick™ EdU-488 Cell Proliferation Assay Kit (Beyotime Biotechnology, Shanghai, China, Cat. No. C0071S). In summary, pre-heated (37 °C) EdU solution (final concentration 10 μM) was introduced to each well, and the cells were incubated for 2 h. The 48-well plates were thereafter taken out, and the cells were rinsed three times with PBS, after which they were fixed with 1 mL of 4% paraformaldehyde for 15 min at ambient temperature. Following fixation, cells underwent three consecutive washes with washing buffer (PBS with 3% BSA) and they were permeabilized for 15 min at ambient temperature using permeabilization buffer (PBS with 0.3% Triton X-100), washed three additional times with washing buffer, and subsequently incubated with the click reaction working solution for 30 min at room temperature. Ultimately, cell nuclei were stained with Hoechst as a counterstain. Cells were visualized with an inverted fluorescent microscope, and the proportion of EdU-positive cells was measured utilizing ImageJ 1.54g software.

### 2.8. Crystal Violet Staining

Cells were inoculated onto 96-well plates for the crystal violet staining. The cell plates were collected at 0, 24, 48, and 72 h following transfection for further examination. The cells were preserved using 4% paraformaldehyde for 15 min, followed by three washings with PBS both prior to and subsequent to fixation. Following this, cells were exposed to a 0.01% crystal violet working solution for 30 min at ambient temperature, after which they were rinsed three times with PBS. Following air-drying in a fume hood, the cells were examined and captured with a light microscope. A semi-quantitative assessment of crystal violet staining outcomes was conducted by introducing 200 μL of 20% glacial acetic acid to each well for dye extraction, followed by measuring the absorbance (OD value) at 490 nm with a microplate reader.

### 2.9. Detection of cAMP Content

After harvesting cultured cells, discard the medium and wash twice with PBS. Harvest cells via trypsin digestion, resuspend in PBS, and transfer to 1.5 mL centrifuge tubes. Perform three repeated freeze–thaw cycles between −20 °C and room temperature to achieve complete cell lysis, then collect supernatants for detection. All subsequent cAMP/PKA content detection operations strictly follow the manufacturer’s instructions (Shanghai Elisa Biotechnology Co., Ltd., Shanghai, China): Equilibrate the microplate at room temperature for 60 min, set standard and sample wells, add corresponding standard (50 μL) or test sample (50 μL) to assigned wells, then add 100 μL HRP-labeled detection antibody to all wells except the blank well. Seal the plate and incubate at 37 °C for 60 min, then perform 5 consecutive wash steps (350 μL wash buffer per well, 1 min incubation per wash). After washing, add equal volumes of substrate A and B (50 μL each) and incubate in the dark at 37 °C for 15 min. Add 50 μL stop solution, then measure OD values at 450 nm within 15 min. Generate a standard curve by plotting standard OD values against their corresponding concentrations, then calculate sample target analyte concentrations via the derived linear regression equation.

### 2.10. cAMP/PKA Signaling Pathway Inhibitor Intervention

SQ22536 and H89 inhibitors were purchased from Selleck Chemicals (Houston, TX, USA). The inhibitor powders were dissolved in freshly opened DMSO to prepare a 200 mM stock solution of SQ22536 and a 50 mM stock solution of H89, respectively, and the prepared stock solutions were stored at −80 °C. Referring to the recommended working concentration range indicated in the official datasheets of the inhibitors, the two stock solutions were diluted with serum-free medium, complete growth medium, and adipogenic induction medium, respectively, to obtain working solutions with different concentration gradients. F3-generation goat intramuscular preadipocytes were seeded into cell culture plates. After 24 h of adherent culture, the medium was replaced with serum-free medium supplemented with corresponding concentrations of SQ22536 or H89, and the cells were further incubated for 3 to 6 h.

Subsequently, cell transfection was performed according to the specific experimental protocol, followed by replacement of the medium with inhibitor-supplemented complete medium or adipogenic induction medium as required. Finally, cell samples were harvested at the pre-determined time points as specified in the experimental design.

### 2.11. Western Blotting

Total protein was extracted from cultured goat intramuscular preadipocytes using 150 μL RIPA lysis buffer supplemented with protease inhibitor and phosphatase inhibitor (both added at a 1:100 volume ratio). Protein concentration was determined using the BCA assay, and equal amounts of protein samples were denatured at 95–100 °C for 10 min.

Protein samples (30 μg per lane) were loaded onto a 12% sodium dodecyl sulfate polyacrylamide gel (SDS-PAGE) for electrophoretic separation: constant voltage 80 V for 30 min in the stacking gel, followed by 120 V for 90 min in the separating gel. After electrophoresis, proteins were transferred to polyvinylidene difluoride (PVDF) membranes using an ice-bath wet transfer system at a constant current of 300 mA for 1 h. Membranes were blocked with 5% skimmed milk for 2 h at room temperature, then incubated with primary antibodies overnight at 4 °C on a shaker: anti-phosphorylated protein kinase A (anti-p-PKA, 1:500, Abclonal, Wuhan, China), anti-total protein kinase A (anti-PKA, 1:1000, Abclonal, Wuhan, China), and anti-β-actin (1:1000, Cell Signaling Technology, Danvers, MA, USA). After washing three times with TBST buffer (10 min each), membranes were incubated with horseradish peroxidase (HRP)-conjugated secondary antibody (1:8000, Abclonal, Wuhan, China) for 1 h at room temperature. Following washing, target protein bands were visualized using an electrochemiluminescence (ECL) detection system. Total protein normalization was used for protein quantification in this study. The optical density value of each protein band was quantitatively measured using ImageJ 1.54g software, and all experiments were performed with 3 independent biological replicates.

### 2.12. Real-Time Fluorescence Quantitative PCR (qPCR)

Total RNA was extracted from 6-well plate-cultured cells using Trizol reagent (TaKaRa, Kyoto, Japan). After air-drying, the RNA pellet was dissolved in DEPC water, and its purity and concentration were determined via spectrophotometric measurement of A260/A280 ratios. RNA was reverse transcribed into cDNA using the RevertAid First Strand cDNA Synthesis Kit (Thermo Fisher Scientific, Waltham, MA, USA). qPCR was performed on a Bio-Rad real-time PCR system with primers listed in Table 1, and UXT was used as the internal reference for normalization of target gene expression, with 3 technical replicates per reaction.

### 2.13. Statistical Analysis

Quantitative PCR (qPCR) data were evaluated via the 2^−ΔΔCt^ method, with all results shown as “mean ± standard error (Mean ± SEM)”. GraphPad Prism 10.1 software was utilized for generating comparative plots among groups. The statistical significance of differences between two groups was evaluated by independent unpaired Student’s *t*-test. For comparisons involving three or more groups, one-way analysis of variance (one-way ANOVA) followed by Tukey’s HSD post hoc multiple comparison test was used for inter-group difference verification. For datasets from inhibitor intervention rescue experiments, two-way analysis of variance (two-way ANOVA) was first performed to assess the main effects of different factors and their interaction effects, and the simple effect differences between pre-defined paired groups under the same treatment condition were verified by Sidak-corrected independent *t*-tests. All variance analyses were performed using SPSS 26.0 software (SPSS Inc., Chicago, IL, USA). Statistical significance was determined at *p* < 0.05, with “*”: *p* < 0.05 (significant difference) and “**”: *p* < 0.01 (very significant difference).

## 3. Results

### 3.1. GPR35 Negatively Regulated Goat Intramuscular Adipocyte Differentiation

To determine whether GPR35 is involved in the differentiation of goat intramuscular adipocytes, we first performed Oil Red O staining to evaluate intramuscular preadipocyte differentiation and visually quantify lipid droplet accumulation. The results demonstrated that both the number and size of lipid droplets increased with induction time at 24 h and 48 h (Figure S1A,B, Supplementary Materials). Concurrently, qPCR analysis revealed that the mRNA expression level of *GPR35* was significantly upregulated by approximately 1.7-fold at 24 h of adipogenic differentiation, and by approximately 1.4-fold at 48 h (Figure S1C,D, Supplementary Materials). These results confirmed that the adipogenic induction system effectively drove intramuscular preadipocyte differentiation and suggested that GPR35 may play a regulatory role during this process.

**Figure 1:**
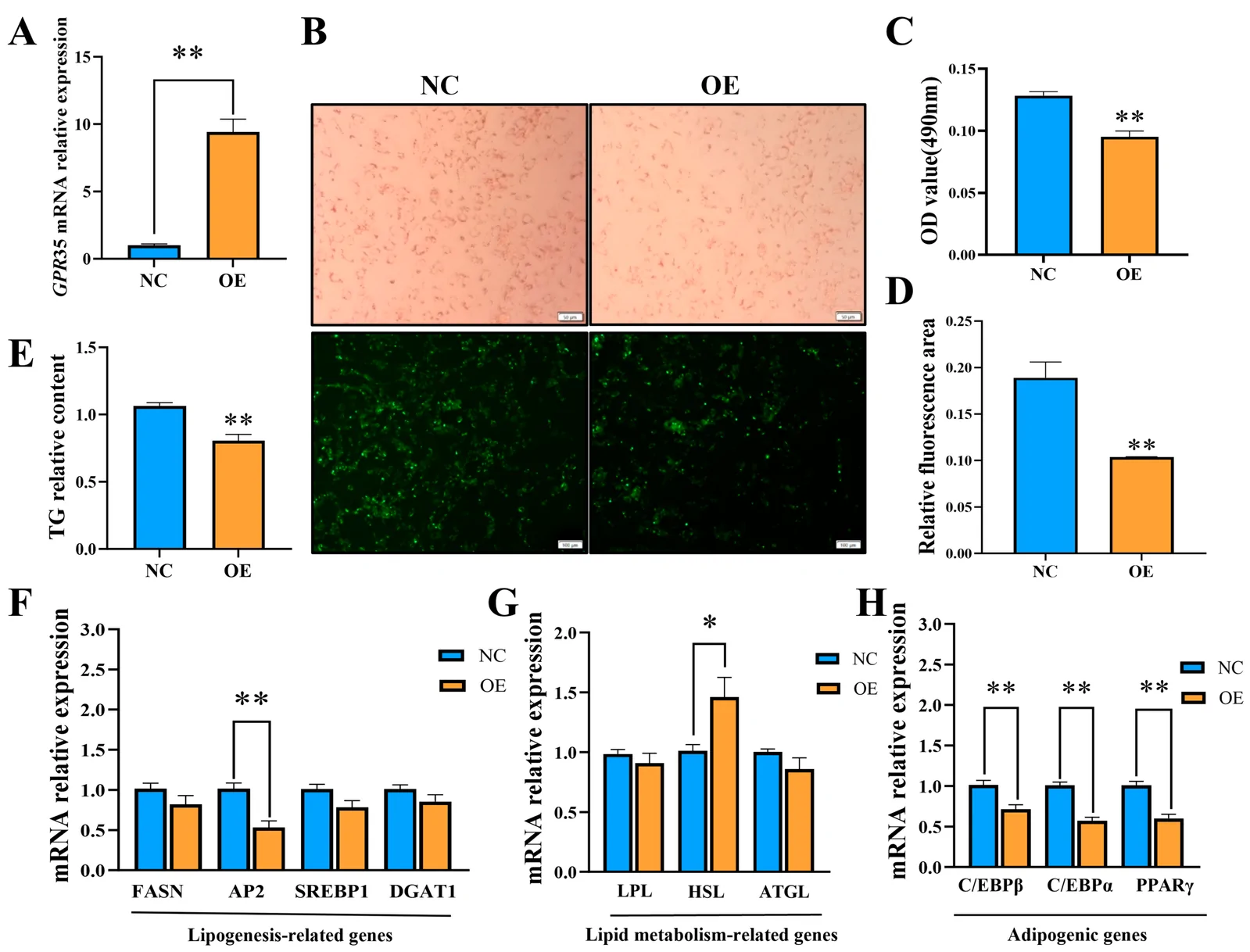
GPR35 overexpression inhibits the adipogenic differentiation of goat intramuscular preadipocytes. (**A**) Relative *GPR35* mRNA expression in goat intramuscular preadipocytes at 48 h after transfection with pEGFP-N1 (NC) or pEGFP-GPR35 (OE), detected by qPCR. (**B**) Representative images of intracellular lipid accumulation stained by Oil Red O (200×) and BODIPY (100×). (**C**) Semi-quantitative analysis of Oil Red O staining: the stained lipid droplets were eluted with isopropanol, and the absorbance was measured at 490 nm. (**D**) Semi-quantitative analysis of BODIPY fluorescence area using ImageJ software. (**E**) Quantification of intracellular triglyceride (TAG) content in NC and GPR35 overexpression groups. Relative mRNA levels of adipogenic marker genes detected by qPCR: (**F**) lipogenesis-related genes; (**G**) lipid metabolism-related genes; (**H**) adipogenic genes. All data are presented as mean ± SEM, *n* = 3. * *p* < 0.05, ** *p* < 0.01.

To investigate the functional role of GPR35 in the adipogenic differentiation of goat intramuscular preadipocytes, we first transfected goat intramuscular preadipocytes with the GPR35 overexpression vector. Compared with the negative control group (NC), the relative mRNA expression of *GPR35* was significantly upregulated by approximately 10-fold (Figure 1A). Subsequently, BODIPY and Oil Red O staining were performed to evaluate intracellular lipid accumulation, and the results revealed that GPR35 overexpression significantly reduced lipid droplet aggregation in goat intramuscular adipocytes (Figure 1B–D). In addition, the quantification of intracellular triglyceride content further confirmed that GPR35 overexpression significantly inhibited the adipogenic differentiation of goat intramuscular preadipocytes (Figure 1E). At the molecular level, GPR35 overexpression significantly downregulated the mRNA levels of adipogenic markers *C/EBPβ*, *C/EBPα*, and *PPARγ*; meanwhile, that of the lipogenesis-related gene *AP2* was significantly downregulated, and the mRNA level of the lipid metabolism-related gene *HSL* was significantly upregulated (Figure 1F–H).

**Figure 2:**
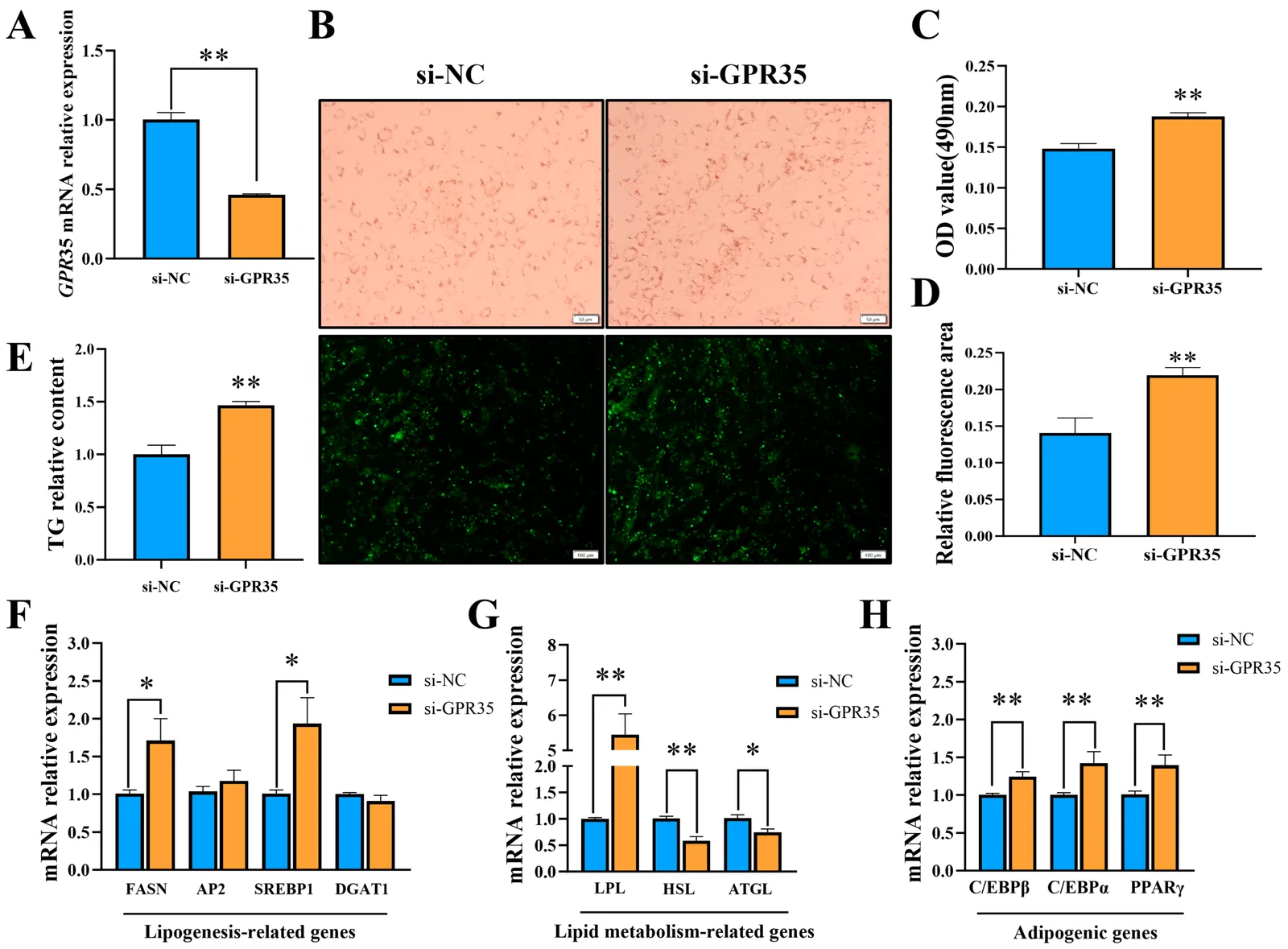
GPR35 knockdown promotes the adipogenic differentiation of goat intramuscular preadipocytes. (**A**) Relative GPR35 *mRNA* expression in goat intramuscular preadipocytes at 48 h after transfection with si-NC or si-GPR35, detected by qPCR. (**B**) Representative images of intracellular lipid accumulation stained by Oil Red O (200×) and BODIPY (100×). (**C**) Semi-quantitative analysis of Oil Red O staining: stained lipid droplets were eluted with isopropanol, and absorbance was measured at 490 nm. (**D**) Semi-quantitative analysis of BODIPY fluorescence area using ImageJ software. (**E**) Quantification of intracellular triglyceride (TAG) content in si-NC and si-GPR35 groups. Relative mRNA levels of adipogenic marker genes detected by qPCR: (**F**) lipogenesis-related genes; (**G**) lipid metabolism-related genes; (**H**) adipogenic genes. All data are presented as mean ± SEM, *n* = 3. * *p* < 0.05, ** *p* < 0.01.

To further investigate the effect of GPR35 on the adipogenic differentiation of goat intramuscular preadipocytes, we transfected goat intramuscular preadipocytes with specific GPR35 siRNA to knock down GPR35 expression. Compared with the negative control (si-NC) group, GPR35 knockdown efficiency was approximately 40% in the si-GPR35 group (Figure 2A). Subsequently, morphological observation, including Oil Red O staining and BODIPY staining, was performed. As expected, the results revealed that si-GPR35 significantly promoted intracellular lipid content and lipid droplet density in goat intramuscular preadipocytes compared with the si-NC group (Figure 2B–D). The intracellular triglyceride (TG) content showed the same trend (Figure 2E). Consistently, the expression of adipocyte differentiation marker genes was detected via qPCR. The results demonstrated that after 48 h of adipogenic differentiation, si-GPR35 significantly upregulated the mRNA expression of *C/EBPβ*, *C/EBPα*, *PPARγ*, and *LPL*, while the mRNA expression of *HSL* and *ATGL* was significantly downregulated (Figure 2F–H). In conclusion, GPR35 acts as a negative regulator of adipogenic development in goat intramuscular preadipocytes and significantly inhibits lipid accumulation inside these cells.

### 3.2. GPR35 Negatively Regulated Goat Intramuscular Adipocyte Proliferation

**Figure 3:**
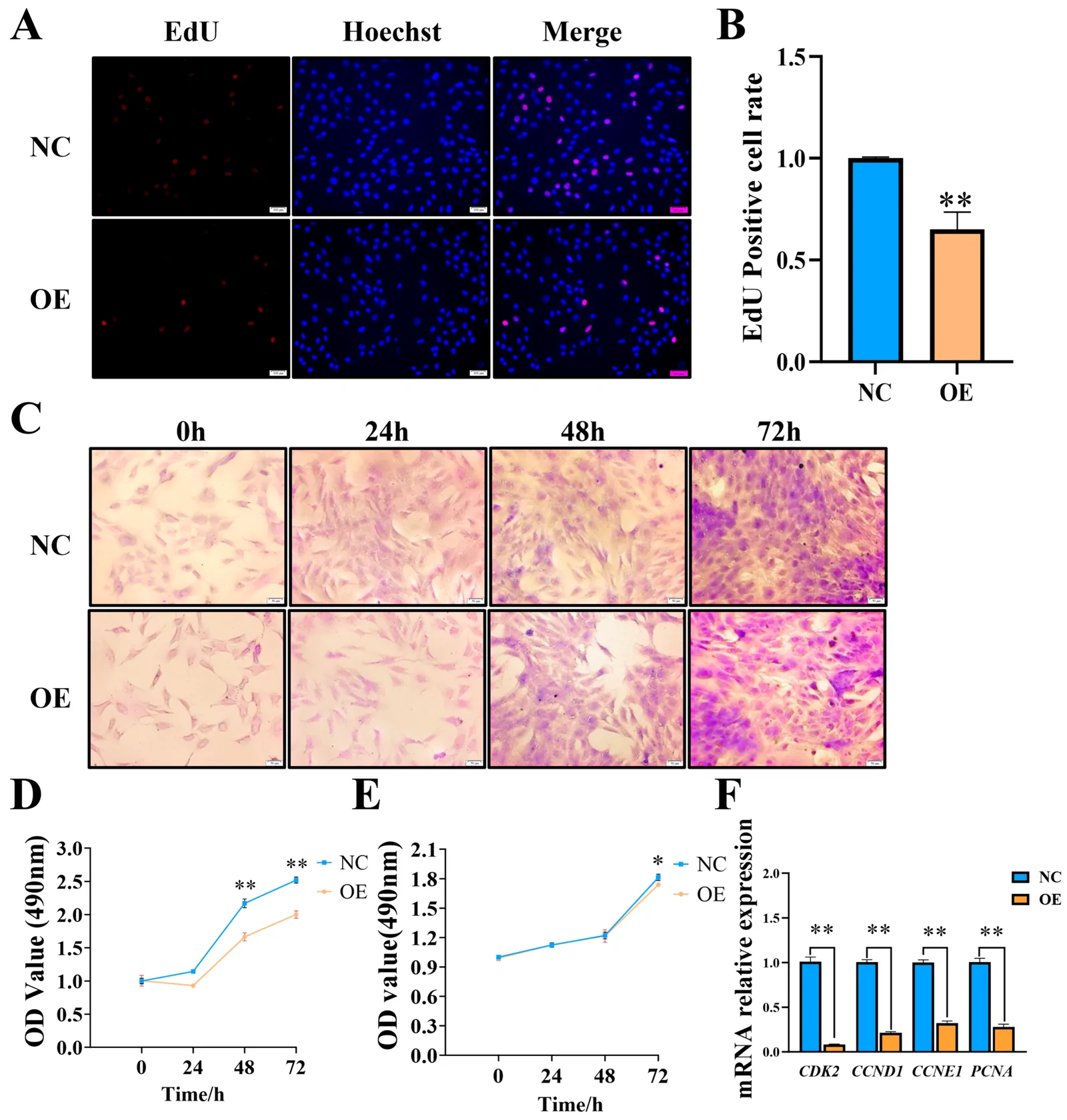
Overexpression of GPR35 inhibits the proliferation of goat intramuscular preadipocytes. (**A**) EdU assay detection of cell proliferation capacity: proliferating cells were labeled with EdU (red), and cell nuclei were counterstained with Hoechst (blue). (**B**) Semi-quantitative statistics of EdU-positive cell proportion in NC and OE groups using ImageJ software. (**C**) Representative images of crystal violet staining showing cell numbers at 0, 24, 48 and 72 h post-transfection. (**D**) Semi-quantitative analysis of crystal violet content by absorbance detection at 490 nm. (**E**) Cell viability at different time points post-transfection was measured via MTT assay. (**F**) Relative mRNA expression levels of cell proliferation marker genes in goat intramuscular preadipocytes transfected with pEGFP-N1 (NC) or pEGFP-GPR35 (OE) detected by qPCR. All data are presented as mean ± SEM, *n* = 3. * *p* < 0.05, ** *p* < 0.01.

Fat deposition relies on both the adipogenic differentiation of mature adipocytes and the clonal expansion of preadipocytes. To evaluate whether GPR35 regulates the proliferation of goat intramuscular preadipocytes, EdU staining was first performed to detect cell proliferation, which revealed that overexpression of GPR35 significantly reduced the EdU positive cell rate (Figure 3A,B). Next, crystal violet staining was performed to assess cell growth at 24, 48 and 72 h after GPR35 transfection. The results demonstrated that the number of cells in the GPR35 overexpression group was significantly reduced at 48 h and 72 h (Figure 3C,D). Meanwhile, MTT assay revealed that GPR35 overexpression significantly suppressed cell viability at 72 h post-transfection compared with the vector group (Figure 3E). In addition, we analyzed the transcriptional levels of cell proliferation-related marker genes. Compared with the negative control, overexpression of GPR35 in goat intramuscular preadipocytes significantly downregulated the mRNA expression of *PCNA*, *CCND1*, *CDK2*, and *CCNE1* (Figure 3F).

**Figure 4:**
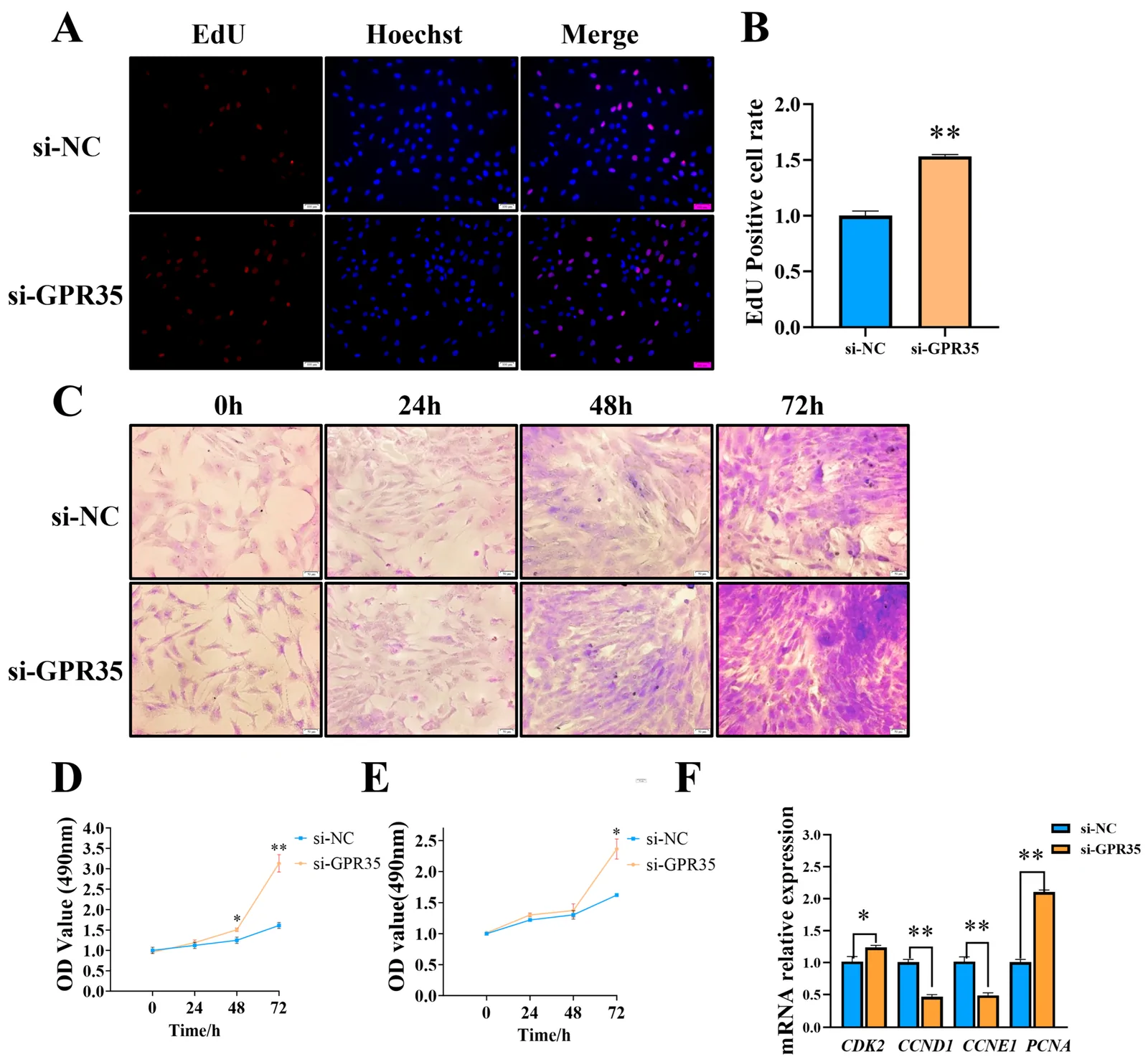
Knockdown of GPR35 promotes the proliferation of goat intramuscular preadipocytes. (**A**) EdU assay detection of cell proliferation capacity: proliferating cells were labeled with EdU (red), and cell nuclei were counterstained with Hoechst (blue). (**B**) Semi-quantitative statistics of EdU-positive cell proportion in si-NC and si-GPR35 using ImageJ software. (**C**) Representative images of crystal violet staining showing cell numbers at 0, 24, 48 and 72 h post-transfection. (**D**) Semi-quantitative analysis of crystal violet content by absorbance detection at 490 nm. (**E**) Cell viability at different time points post-transfection was measured via MTT assay. (**F**) Relative mRNA expression levels of cell proliferation marker genes *CDK2*, *CCND1*, *CCNE1*, and *PCNA* in two groups detected by qPCR. All data are presented as mean ± SEM, *n* = 3. * *p* < 0.05, ** *p* < 0.01.

Loss-of-function experiments were performed to further investigate the role of GPR35 in goat intramuscular preadipocyte proliferation. The EdU detection results revealed that the positive cell rate significantly increased after knockdown of GPR35 for 48 h (Figure 4A,B). Furthermore, the crystal violet staining and MTT assay demonstrated that si-GPR35 significantly promoted the number and viability of goat intramuscular preadipocytes at 48 h and 72 h (Figure 4C–E). Subsequently, qPCR was performed to detect the mRNA expression levels of genes associated with cell proliferation, including *PCNA*, *CCND1*, *CDK2*, and *CCNE1*. The results showed that the mRNA expression levels of *CDK2* and *PCNA* were significantly upregulated, while those of *CCND1* and *CCNE1* were significantly downregulated (Figure 4F). Taken together, GPR35 functions as a negative regulator: it reduces the number of clonally expanding cells in the initial proliferation phase of intramuscular preadipocytes, while simultaneously inhibiting their subsequent directed differentiation into mature adipocytes and reducing lipid accumulation.

### 3.3. Identification of the Downstream Signal Pathways of GPR35

**Figure 5:**
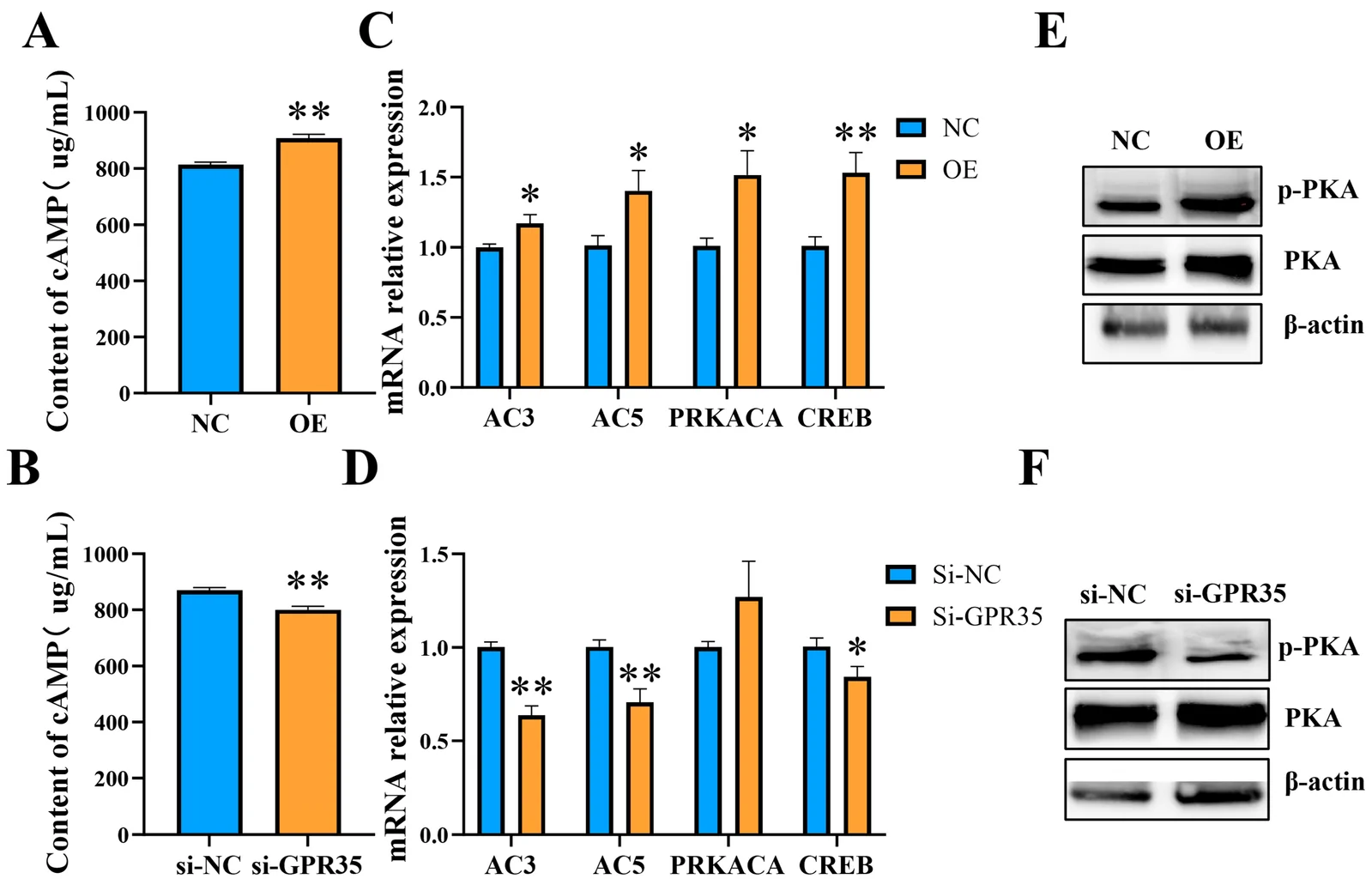
GPR35 activates the cAMP/PKA signaling pathway in goat intramuscular preadipocytes. (**A**) Intracellular cAMP concentration in cells after 48 h of GPR35 overexpression transfection, detected by ELISA kit. (**B**) Intracellular cAMP concentration in cells after si-GPR35/si-NC transfection, detected by ELISA kit. (**C**) Relative mRNA levels of core cAMP/PKA pathway genes *AC3*, *AC5*, *PRKACA*, and *CREB* in overexpression groups and control groups. T-shaped lines in the plot denote standard error of the mean (SEM) from at least three independent biological replicates. (**D**) Relative mRNA levels of core cAMP/PKA pathway genes *AC3*, *AC5*, *PRKACA*, and *CREB* in si-GPR35 group. (**E**) Western blot analysis of p-PKA and PKA protein expression in OE and NC groups, with β-actin as the internal loading control. (**F**) Western blot analysis of p-PKA and PKA protein expression in si-GPR35 and si-NC groups, with β-actin as the internal loading control. All data are presented as mean ± SEM, *n* = 3. * *p* < 0.05, ** *p* < 0.01.

The cAMP/PKA signaling pathway is the most classic downstream pathway of the G protein-coupled receptor (GPCR) family and it has been reported to be crucial for differentiation and proliferation of adipocytes [29,30]. Members of the GPR family may regulate signal transduction through different mechanisms in the cAMP pathway [31]. To verify the close relationship between GPR35 and the cAMP signaling pathway, we conducted gain-of-function and loss-of-function experiments. ELISA assay results indicated that GPR35 overexpression significantly elevated intracellular cAMP levels, whereas GPR35 knockdown reduced cAMP levels (Figure 5A,D). Further qPCR analysis revealed that GPR35 overexpression significantly upregulated the mRNA levels of key marker genes of the cAMP/PKA signaling pathway, including *AC3*, *AC5*, *PRKACA* and *CREB* (Figure 5B). Conversely, GPR35 knockdown reduced intracellular cAMP levels and significantly downregulated the mRNA levels of *AC3*, *AC5*, *PRKACA*, and *CREB* (Figure 5E). Meanwhile, Western blot was performed to detect PKA phosphorylation levels. Compared with the control group, the phosphorylation level of PKA significantly increased after overexpression of GPR35, while it was significantly reduced after GPR35 knockdown, indicating that GPR35 can activate the cAMP/PKA signaling pathway in goat intramuscular adipocytes (Figure 5C,F).

### 3.4. Inhibiting the cAMP/PKA Signaling Pathway Can Restore the Differentiation Inhibition Phenomenon Observed in Goat Muscle Preadipocytes Due to GPR35 Overexpression

To further elucidate the downstream molecular mechanism by which GPR35 regulates adipogenic differentiation of goat intramuscular preadipocytes and clarify the mediating role of the cAMP/PKA signaling pathway in this process, GPR35-overexpressing goat intramuscular preadipocytes were treated with the cAMP inhibitor SQ22536 or the PKA-specific inhibitor H89 prior to adipogenic induction. To eliminate the interference of inhibitor cytotoxicity on experimental results, we first assessed cell viability after treatment with different concentrations of inhibitors via MTT assay, and verified the pathway blocking efficiency of the inhibitors by detecting PKA phosphorylation levels via Western blot, to finally determine the optimal working concentrations of SQ22536 and H89. The results showed that when the concentration of SQ22536 was 100 μM, it had no effect on cell viability. However, when the concentration was 150 μM, it significantly increased cell viability, and both concentrations significantly inhibited PKA phosphorylation levels (Figure S2A,B). Similarly, H89 had no effect on cell viability at a concentration of 2.5 μM, but was able to inhibit PKA phosphorylation levels. At a concentration of 10 μM, it significantly increased cell viability and also inhibited PKA phosphorylation levels (Figure S2C,D).

**Figure 6:**
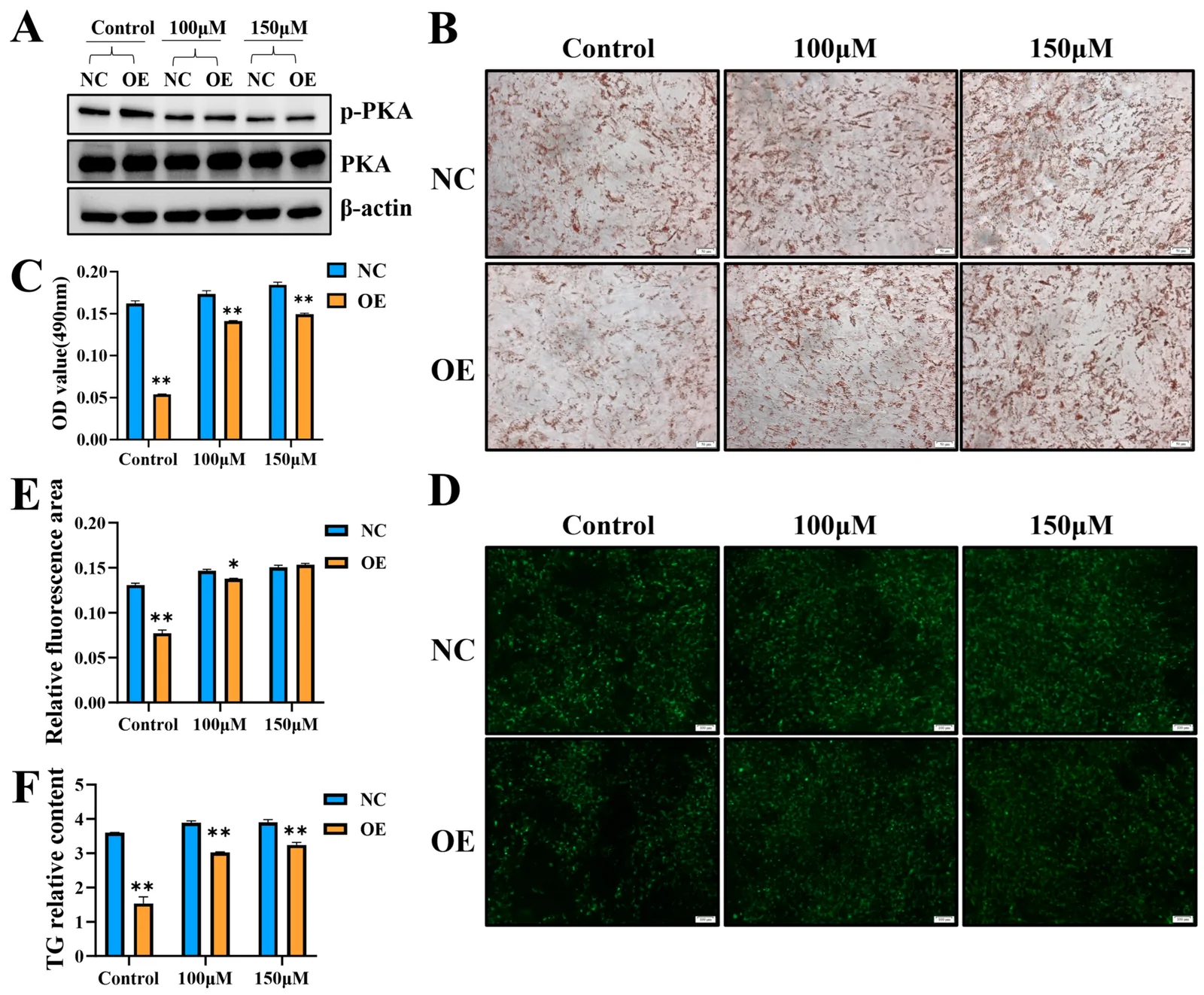
The cAMP inhibitor SQ22536 alleviates the inhibitory effect of GPR35 on adipogenic differentiation of goat intramuscular preadipocytes. (**A**) SQ22536 rescues the regulatory effect of GPR35 overexpression on cAMP/PKA signaling pathway: protein expression levels of PKA and p-PKA detected by Western blot. (**B**) Representative images of Oil Red O staining. (**C**) Semi-quantitative analysis of Oil Red O staining at 490 nm absorbance. (**D**) Representative images of BODIPY staining. (**E**) Semi-quantitative analysis of BODIPY fluorescence area. (**F**) Quantification of intracellular triglyceride (TAG) content in the NC and OE groups with or without SQ22536 treatment. All data are presented as mean ± SEM, *n* = 3. * *p* < 0.05, ** *p* < 0.01.

GPR35-overexpressing intramuscular preadipocytes were treated with 100 μM or 150 μM of SQ22536. The results demonstrated that GPR35 overexpression significantly promoted PKA activation, and this activation was significantly attenuated by SQ22536 treatment (Figure 6A). Subsequently, morphological staining and TG detection revealed that SQ22536 treatment reversed the inhibition of lipid accumulation induced by GPR35 overexpression (Figure 6B–F). Consistent with the phenotype, GPR35 overexpression significantly downregulated the mRNA expression levels of *C/EBPα*, *C/EBPβ*, *PPARγ*, *LPL*, *AP2*, and *SREBP1* (Figure S3A–I, Supplementary Materials). After SQ22536 treatment, the downregulation of these genes was partially reversed. Moreover, overexpression of GPR35 significantly upregulated the expression level of *HSL*. After SQ22536 treatment, the inhibitory effect of GPR35 on adipogenic differentiation was significantly alleviated (Figure S3F, Supplementary Materials). Taken together, these results establish a direct functional link between GPR35, cAMP/PKA pathway activation, and the regulation of adipogenic gene expression and lipid accumulation in goat intramuscular preadipocytes.

**Figure 7:**
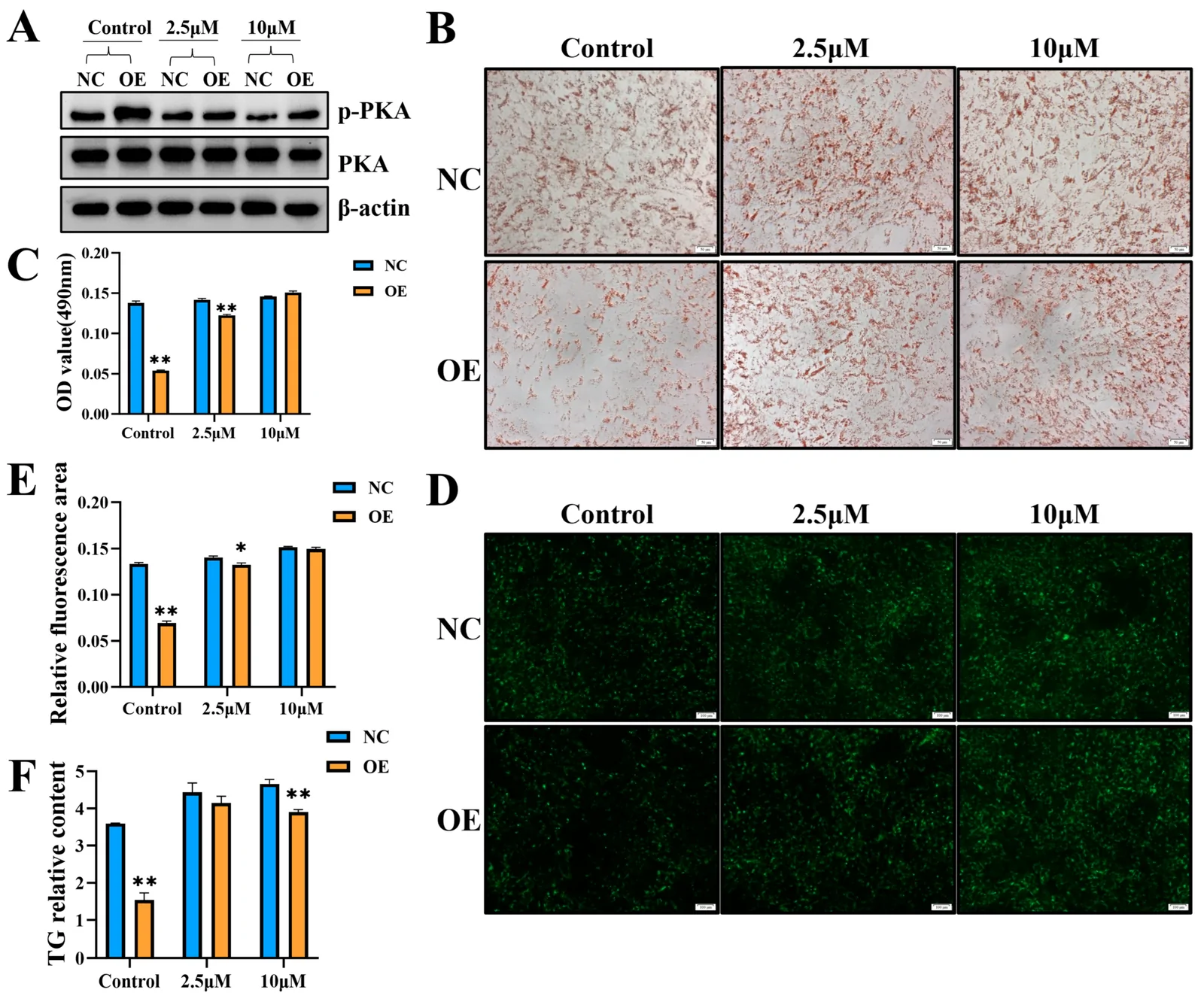
The PKA-specific inhibitor H89 alleviates the inhibitory effect of GPR35 on adipogenic differentiation of goat intramuscular preadipocytes. (**A**) H89 rescues the regulatory effect of GPR35 overexpression on cAMP/PKA signaling pathway: protein expression levels of PKA and p-PKA detected by Western blot. (**B**) Representative images of Oil Red O staining. (**C**) Semi-quantitative analysis of Oil Red O staining at 490 nm absorbance. (**D**) Representative images of BODIPY staining. (**E**) Semi-quantitative analysis of BODIPY fluorescence area. (**F**) Quantification of intracellular triglyceride (TAG) content in the NC and GPR35 overexpression groups with or without H89 treatment. All data are presented as mean ± SEM, *n* = 3. * *p* < 0.05, ** *p* < 0.01.

Meanwhile, GPR35-overexpressing intramuscular preadipocytes were pretreated with 2.5 μM or 10 μM H89, and the activation of the cAMP/PKA pathway was detected by Western blot. The results demonstrated that overexpression of GPR35 increased the phosphorylation level of PKA (Figure 7A). However, after H89 treatment, the ability of GPR35 to upregulate PKA phosphorylation was significantly reduced. Similar to SQ22536, H89 reversed the inhibitory effect of GPR35 overexpression on lipid accumulation in goat intramuscular preadipocytes, and the inhibitory effects of GPR35 overexpression on the mRNA expression of *C/EBPα*, *C/EBPβ*, *PPARγ*, *LPL*, *AP2*, *DGAT1*, *SREBP1*, and *FASN* were partially reversed (Figure 7B–F and Figure S4A–I, Supplementary Materials). In contrast, GPR35 overexpression significantly increased the mRNA level of *HSL*, while H89 significantly attenuated this upregulation (Figure S4F, Supplementary Materials). In conclusion, GPR35 inhibits the differentiation of goat intramuscular preadipocytes by activating the cAMP/PKA signaling pathway.

### 3.5. Inhibition of the cAMP/PKA Signaling Pathway Can Restore GPR35-Overexpression-Caused Proliferation Restraint in Goat Intramuscular Preadipocytes

**Figure 8:**
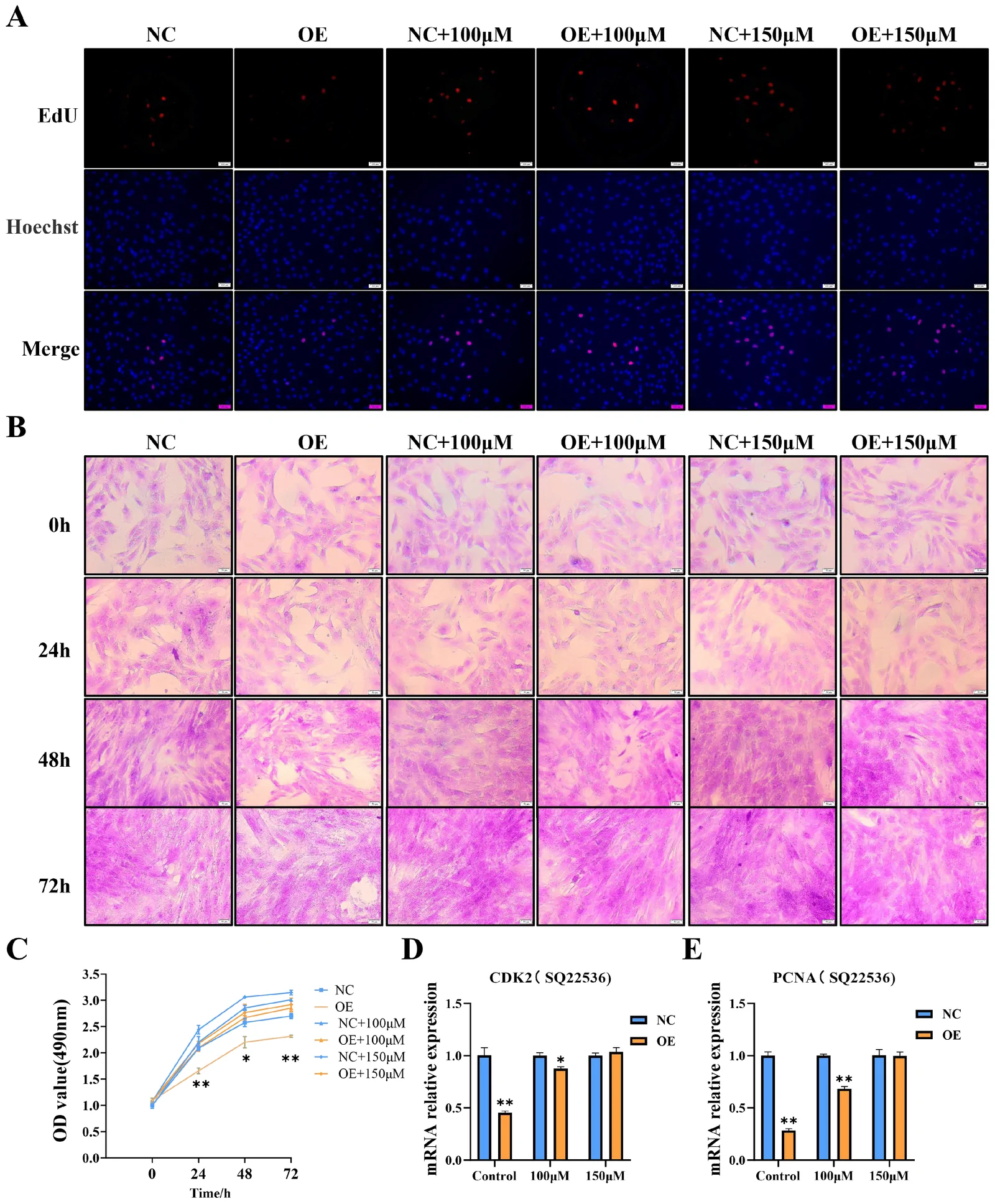
The cAMP inhibitor SQ22536 alleviates the inhibitory effect of GPR35 on the proliferation of goat intramuscular preadipocytes. (**A**) EdU assay detection of cell proliferation: proliferating cells were labeled with EdU (red), and cell nuclei were counterstained with Hoechst (blue). (**B**) Representative images of crystal violet staining. (**C**) Cell viability at indicated time points post-treatment was measured via MTT assay. T-shaped lines in the plot denote standard error of the mean (SEM) from at least three independent biological replicates. (**D**,**E**) Relative mRNA expression levels of cell proliferation marker genes *CDK2* and *PCNA* in the NC and OE groups with or without SQ22536 treatment, detected by qPCR. All data are presented as mean ± SEM, *n* = 3. * *p* < 0.05, ** *p* < 0.01.

In order to verify whether the cAMP/PKA signaling pathway mediates the effect of GPR35 on proliferation of goat intramuscular preadipocytes, we treated GPR35-overexpressing cells with SQ22536 (100 μM or 150 μM) during cell proliferation. The results of Western blotting showed that the 100 μM group had higher p-PKA levels than the 150 μM group and lower levels than the control group (Figure S5A). EdU staining analysis also indicated that the number of EdU-labelled cells was decreased in the OE group compared with the NC group. However, after the addition of SQ22536, the difference in the number of EdU-positive cells between the GPR35 overexpression group and the control group was abolished (Figure 8A and Figure S5B, Supplementary Materials). Subsequently, we performed crystal violet staining and semi-quantitative experiments, and overexpression of GPR35 significantly reduced the number of cells; however, when combined with SQ22536 treatment, this reduction in cell number was largely reversed (Figure 8B and Figure S5C, Supplementary Materials). Meanwhile, cell viability was assessed by MTT assay, and GPR35 overexpression significantly inhibited cell proliferation after 24 h to 72 h transfection, but this effect could be reversed by SQ22536 (Figure 8C). Inhibition of the cAMP/PKA signaling pathway with SQ22536 reversed the downregulation of *CDK2* and *PCNA* induced by GPR35 overexpression (Figure 8D,E).

**Figure 9:**
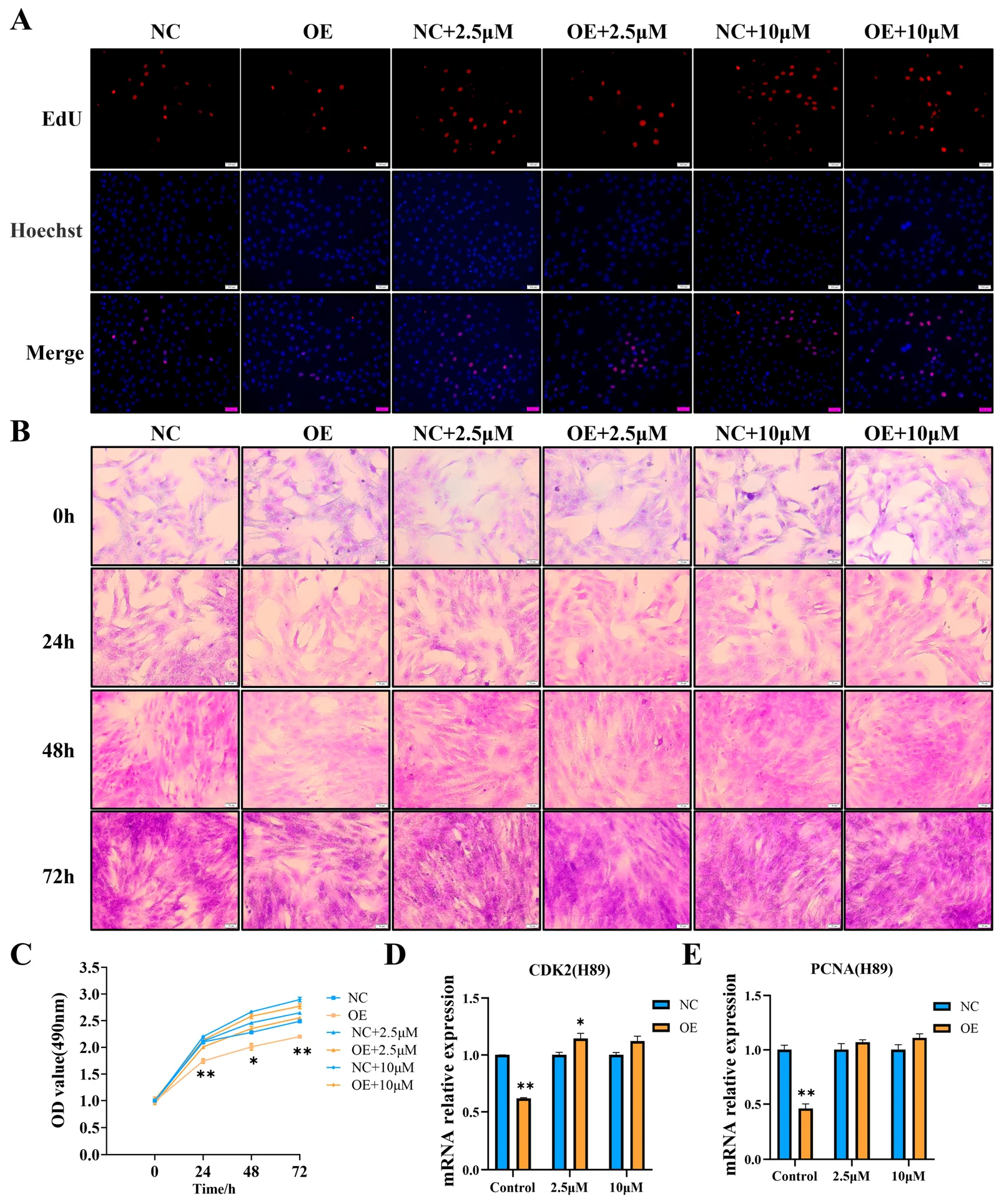
The PKA-specific inhibitor H89 alleviates the inhibitory effect of GPR35 on the proliferation of goat intramuscular preadipocytes. (**A**) EdU assay detection of cell proliferation: proliferating cells were labeled with EdU (red), and cell nuclei were counterstained with Hoechst (blue). (**B**) Representative images of crystal violet staining. (**C**) Cell viability at indicated time points post-treatment was measured via MTT assay. T-shaped lines in the plot denote standard error of the mean (SEM) from at least three independent biological replicates. (**D**,**E**) Relative mRNA expression levels of cell proliferation marker genes *CDK2* and *PCNA* in the NC and OE groups with or without H89 treatment, detected by qPCR. All data are presented as mean ± SEM, *n* = 3. * *p* < 0.05, ** *p* < 0.01.

Similarly, the PKA inhibitor H89 (2.5 μM or 10 μM) was used to test whether the cAMP/PKA pathway mediates the suppressive effect of GPR35 on preadipocyte proliferation. H89 treatment exerted similar effects to SQ22536 in restoring cell proliferation. The 2.5 μM group had higher p-PKA levels than the 10 μM group and significantly lower levels than the control group (Figure S5D). EdU analysis, crystal violet staining and MTT assays collectively showed that H89 treatment could reverse the inhibition of cell proliferation induced by GPR35 overexpression (Figure 9A–C and Figure S5E,F, Supplementary Materials). It has been confirmed that the GPR35-mediated proliferation inhibition is dependent on PKA activity, and the interference caused by the potential non-specific effects of SQ22536 treatment alone has been excluded. In addition, the mRNA levels of *PCNA* and *CDK2* were remarkably downregulated in GPR35-overexpressing cells, which could be reversed to normal levels by H89 treatment (Figure 9D,E). Taken together, these findings strongly suggest that GPR35 inhibits the proliferation of goat intramuscular preadipocytes by activating the cAMP/PKA signaling pathway.

## 4. Discussion

Intramuscular fat deposition is cooperatively regulated by multiple factors including genetic background, nutrition level, gender characteristics and feeding management, and has extremely high potential for precision targeted intervention [32,33]. Therefore, screening key functional candidate genes governing intramuscular fat deposition serves as the core prerequisite for realizing the directional improvement of livestock meat quality traits. Accumulating studies have confirmed that GPR35 is widely involved in the regulation of lipid metabolism, plays a pivotal role in lipid storage and energy homeostasis maintenance in adipose tissue, and serves as a potential therapeutic target for diseases related to lipid metabolism disorders [24,26,34,35]. In the research of Metabolic Dysfunction-Associated Steatotic Liver Disease (MASLD), GPR35 can promote liver injury repair and maintain phospholipid homeostasis. In contrast, GPR35 deficiency leads to excessive lipid accumulation and accelerates the progression of MASLD [34]. Similarly, Wu et al. demonstrated that both global knockout and intestinal epithelium-specific knockout of GPR35 exacerbate high-fat-diet-induced metabolic disorders and hepatic steatosis [36]. The results of temporal expression detection in the primary in vitro culture system of this study showed that the mRNA level of *GPR35* was upregulated by approximately 1.7-fold at 24 h and by approximately 1.4-fold at 48 h during goat intramuscular preadipocyte differentiation. This transient upregulation pattern at the early stage of differentiation is completely consistent with the “brake mechanism” characteristics of reported negative regulators of adipogenesis [37]. Subsequent functional verification results further confirmed this regulatory feature: under the same in vitro culture conditions, GPR35 overexpression significantly reduced the content and accumulation level of lipid droplets in adipocytes, while GPR35 knockdown significantly promoted lipid droplet accumulation. Furthermore, the regulatory effect of GPR35 on adipocyte differentiation depends on the cascade transcriptional regulatory network composed of core adipogenic transcription factors C/EBPα, PPARγ, and C/EBPβ [38,39,40,41,42].

The core factors determining intramuscular fat (IMF) deposition are the number of intramuscular preadipocytes and their adipogenic differentiation capacity [38]. However, GPR35 exerts significant cell-type-specific regulatory effects on cell proliferation [43,44]. GPR35 can promote vascular endothelial cell proliferation and participate in vascular remodeling processes [45]. GPR35 is also highly expressed in multiple tumors including gastric cancer, breast cancer and non-small-cell lung cancer, and the highly expressed GPR35 drives malignant proliferation of tumor cells, presenting a distinct oncogenic function [46,47,48]. Notably, this study found that GPR35, as a negative regulator of adipocyte differentiation, can simultaneously inhibit the differentiation and proliferation of goat intramuscular preadipocytes. Under the in vitro experimental system, methods including EdU assay, crystal violet staining, MTT assay and qPCR were used to investigate the effect of GPR35 on the proliferation of goat intramuscular adipocytes. The results showed that GPR35 overexpression significantly reduced the proportion of EdU-positive cells, the total number of growing cells was markedly decreased at 48 h and 72 h post-transfection, and cell viability was also significantly inhibited at 72 h after transfection. qPCR results showed that GPR35 overexpression significantly downregulated the mRNA levels of positive proliferation regulators, including *CDK2*, *CCND1*, *CCNE1*, and *PCNA*. However, the expression changes of *CCND1* and *CCNE1* did not exhibit the expected trend in the GPR35 knockdown experiment, which may be a secondary effect of the coordinated regulation of multiple downstream pathways. As a multifunctional signaling hub, GPR35 has been reported to mediate multiple proliferation-related signaling cascades, including the Src/Erk/Akt axis and Wnt/β-catenin pathway, and exerts pro-proliferative effects in other cell types [49,50,51,52]. Nevertheless, the inhibitory effect of GPR35 on cell proliferation in goat intramuscular preadipocytes is predominantly mediated by the cAMP/PKA signaling pathway.

Adipocyte differentiation involves multi-level regulation of signaling, transcription and metabolic networks [53]. As a member of the GPCR superfamily, G protein-coupled receptor 35 (GPR35) can interact with intracellular heterotrimeric G proteins (comprising Gα, Gβ, and Gγ subunits), and the downstream functional outputs of different G protein subunits have been confirmed to exhibit clear functional diversification [20,54]. Published studies have verified that GPR35 specifically couples to the Gαs subunit to promote the synthesis of the intracellular second messenger cAMP, thereby activating the classical downstream cAMP/PKA signaling cascade [19,55]; activated PKA directly enhances the catalytic activity of hormone-sensitive lipase (HSL), a key lipolytic enzyme, via phosphorylation modification [56]. Using in vitro cultured primary goat intramuscular preadipocytes as the experimental model, this study found that GPR35 activates the classical PKA phosphorylation-mediated lipolytic pathway by regulating intracellular cAMP levels, and significantly modulates the mRNA level of *HSL*. These results are highly consistent with the previously reported molecular mechanisms. Notably, the cAMP/PKA pathway has a dual regulatory effect on adipocyte differentiation and proliferation. High-concentration cAMP can simultaneously inhibit the differentiation and proliferation of preadipocytes by activating PKA, thereby reducing fat deposition [57,58,59,60]. This experiment preliminarily verified that GPR35 participates in the regulation of intramuscular fat (IMF) deposition via the cAMP/PKA signaling pathway, through in vitro detection of cAMP content in the GPR35 overexpression group and knockdown group, the mRNA expression levels of pathway-related genes (*AC3*, *AC5*, *PRKACA*, and *CREB*), as well as the protein expression levels of PKA and p-PKA.

However, GPR35 remains a poorly characterized GPCR. As an orphan receptor, although it has been confirmed to interact with ligands such as CXCL17 and kynurenic acid (KYNA), existing studies indicate that these ligands exhibit low binding affinity to GPR35, and their regulatory effects present significant species specificity and context dependence [61,62,63]. Therefore, to directly verify whether the inhibitory effects of GPR35 on the maturation, differentiation and early proliferation of intramuscular preadipocytes, and its subsequent modulation of IMF deposition, are dependent on the activation of the cAMP/PKA pathway, we pretreated cells with the adenylate cyclase inhibitor SQ22536 and the PKA-specific inhibitor H89 under in vitro conditions. In our study, after treating cells with two inhibitors, both the two phenotypes (reduced lipid droplet accumulation and decreased intracellular triglyceride (TG) content) induced by GPR35 overexpression were significantly reversed. Meanwhile, the mRNA levels of adipocyte differentiation marker genes were restored to varying degrees. Furthermore, inhibitor intervention could significantly rescue the GPR35 overexpression-mediated proliferation-suppressive effect in goat intramuscular preadipocytes and abrogate the mRNA downregulation trend of cell proliferation marker genes. In the in vitro primary culture system, GPR35 exerts a negative regulatory effect on lipid deposition in goat intramuscular preadipocytes mainly via the cAMP/PKA signaling pathway. However, the in-depth regulatory relationship between PKA and its downstream functional targets remains to be systematically explored in subsequent studies. The functional validation of the signaling pathway in this study is primarily based on pharmacological inhibitor intervention experiments. The selected SQ22536 and H89 are widely recognized classic tool reagents in the field of cAMP/PKA signaling pathway research. Nevertheless, the potential non-specific off-target effects of these two agents under the experimental conditions employed in this study cannot be completely excluded. The targeted regulation of goat intramuscular fat deposition and the final directional improvement of meat quality traits based on the molecular pathway revealed in this study still require further in-depth investigation.

## 5. Conclusions

This study first establishes the temporal expression profile of GPR35 in goat intramuscular preadipocytes and confirms its essential involvement in the adipogenic differentiation process of intramuscular adipocytes. GPR35 is identified as a negative regulator of adipogenesis: GPR35 overexpression significantly suppresses cell proliferation, differentiation and lipid deposition, while GPR35 silencing effectively promotes the maturation of intramuscular preadipocytes into adipocytes. Mechanistically, GPR35 activates the cAMP/PKA signaling pathway by elevating intracellular cAMP content, enhancing PKA phosphorylation level, and upregulating the transcription of key pathway genes (*AC3*/*AC5*/*PRKACA*/*CREB*). Notably, pharmacological inhibition of this pathway with SQ22536 and H89 can completely reverse the suppressive effects of GPR35 on lipid droplet accumulation, triglyceride content, and the expression of marker genes related to differentiation and proliferation. In conclusion, through gain- and loss-of-function experiments combined with pathway inhibitor validation, this study demonstrates that the cAMP/PKA signaling pathway is a critical factor for the negative regulatory function of GPR35 on the proliferation and adipogenesis of goat intramuscular preadipocytes under the in vitro culture system.

## Figures and Tables

**Table 1:** Primer information.

Primer Names	Primer Sequence (5′→3′) *	Size (bp)	Tm (*T*/°C)	Purpose
*GPR35*	S: 5′ CGCCATCTGCTACTACTTCAT 3′ A: 5′ CAGACTGTCCTTGCTCTTGTG 3′	97	60	qPCR
*AC3*	S: 5′ GCTGTCTGCTCCATCCTCATGG 3′ A: 5′ CCATGGTGCTCTGGGAGATGTG 3′	235	62	qPCR
*AC5*	S: 5′ CTCTTTGACAATGCCGACCTC 3′ A: 5′ ACGCACTCGCACGACTGAT 3′	350	62	qPCR
*PRKACA*	S: 5′ CCAAGAAGGGCAGTGAGCAAG 3′ A: 5′ GTTCAAACTGATCCAAGTGGGC 3′	117	61	qPCR
*CREB*	S: 5′ AGTCCAGACAGTTCAGATTTCA 3′ A: 5′ AGGAAGGCCTCCTTGAAAGAAT 3′	113	59	qPCR
*CCNE1*	S: 5′ CTCCCTGATTCCCACACCTG 3′ A: 5′ CATAAGATGCTTGTCCCTCA 3′	193	60	qPCR
*PCNA*	S: 5′ AGTGGAGAACTTGGAAATGGAA 3′ A: 5′ GAGACAGTGGAGTGGCTTTTGT 3′	154	60	qPCR
*CCND1*	S: 5′ TGAACTACCTGGACCGCT 3′ A: 5′ CAGGTTCCACTTGAGTTTGT 3′	212	58	qPCR
*CDK2*	S: 5′ GCCAGGAGTTACTTCTATGC 3′ A: 5′ TGGAAGAAAGGGTGAGCC 3′	180	56	qPCR
*TBP*	S: 5′ AACAGCCTCCCACCTTATGC 3′ A: 5′ TGCTGCTCCTCCAAAATAGAC 3′	155	60	qPCR
*UXT*	S: 5′ GCAAGTGGATTTGGGCTGTAAC 3′ A: 5′ ATGGAGTCCTTGGTGAGGTTGT 3′	173	60	qPCR
*ATGL*	S: 5′ CAAGGAGACGACGTGGAACA 3′ A: 5′ CATAGATGTGCGTGGCGTTG 3′	125	60	qPCR
*AP*2	S: 5′ GAAAGAAGTGGGTGTGGGCT 3′ A: 5′ TGGTGGTAGTGACACCGTTC 3′	317	60	qPCR
*C/EBPα*	S: 5′ GGCATCTGCGAACACGAGAC 3′ A: 5′ CAGGAACTCGTCGTTGAAGG 3′	75	60	qPCR
*C/EBPβ*	S: 5′ CAAGAAGACGGTGGACAAGCAC 3′ A: 5′ CTTGAACAAGTTCCGCAGGGT 3′	208	62	qPCR
*FASN*	S: 5′ TTGTGGGCTTGGTGAACTGT 3′ A: 5′ GGGCTTGTCTTGTTCCAGTAGG 3′	215	60	qPCR
*LPL*	S: 5′ GGTGACAGGAATGTATGAGAGTTGG 3′ A: 5′ CCCAAGGCTGTATCCCAAGAG 3′	235	60	qPCR
*SREBP1*	S: 5′ GGTTTCCAGAGGGACTTGAGTG 3′ A: 5′ CCCTCCTCCTCAGGCTACGAT 3′	157	62	qPCR
*DGAT1*	S: 5′ GTGGCTTCAGCAACTACCGT 3′ A: 5′ GCCACAATGACCAGGCACAG 3′	181	62	qPCR
*HSL*	S: 5′ AGGGTCATTGCCGACTTCC 3′ A: 5′ GTCTCGTTGCGTTTGTAGTGC 3′	161	60	qPCR
*PPARγ*	S: 5′ AAGCGTCAGGGTTCCACTATG 3′ A: 5′ GAACCTGATGGCGTTATGAGAC 3′	197	60	qPCR

* S: Sense primer; A: Antisense primer.

## Data Availability

The original contributions presented in this study are included in the article/Supplementary Material. Further inquiries can be directed to the corresponding author.

## Supplementary Materials

The following supporting information can be downloaded at: https://www.mdpi.com/article/10.3390/ani16162560/s1, Supplemental Figure S1: Temporal expression profile of GPR35 in intramuscular adipocytes; Supplemental Figure S2: Screening of the optimal effective concentrations of cAMP inhibitor SQ22536 and PKA phosphorylation inhibitor H89 in goat intramuscular preadipocytes; Supplemental Figure S3: SQ22536 alleviates the inhibitory effect of GPR35 on the adipogenic differentiation of goat intramuscular preadipocytes; Supplemental Figure S4: H89 alleviates the inhibitory effect of GPR35 on the adipogenic differentiation of goat intramuscular preadipocytes; Supplemental Figure S5: Inhibition of the cAMP/PKA signaling pathway restores GPR35 overexpression-induced proliferation restraint in goat intramuscular preadipocytes.
