# Supplementary material for: GPR35 Mediates the Proliferation and Adipogenesis of Goat Intramuscular Preadipocytes Through the cAMP/PKA Signaling Pathway
Source: Animals (Basel). 2026 Aug 17;16(16):2560. doi: 10.3390/ani16162560 (PMC13508854; doi:10.3390/ani16162560)
Supplement: Supplementary file 1 [file animals-16-02560-s001.zip › animals-4449629-supplementary.pdf]

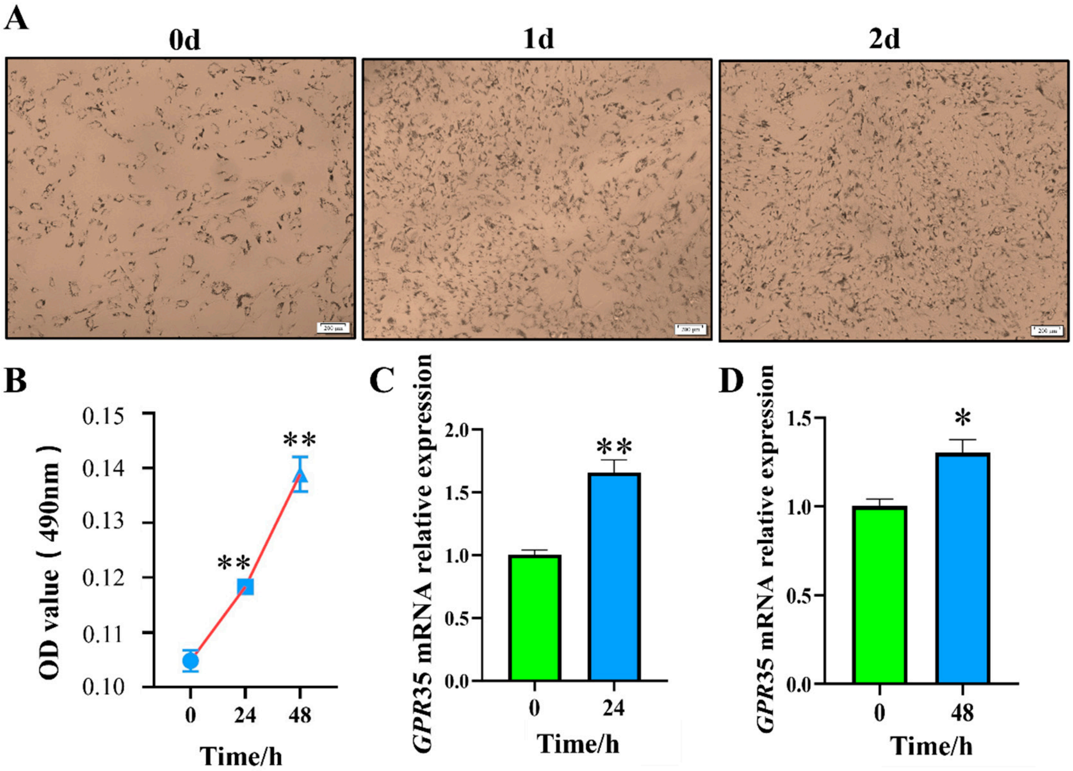

Figure S1. Temporal expression profile of GPR35 in intramuscular adipocytes. (A) Representative images of Oil Red O staining at days 0, 1, and 2 after adipogenic induction. (B) Quantitative results of Oil Red O staining during the sequential differentiation of goat intramuscular preadipocytes. (C) GPR35 mRNA expression in goat intramuscular preadipocytes after 24 h of adipogenic induction. (D) GPR35 mRNA expression in goat intramuscular preadipocytes after 48 h of adipogenic induction. All data are presented as mean ± SEM, $n = 3$. * $p < 0.05$, ** $p < 0.01$.

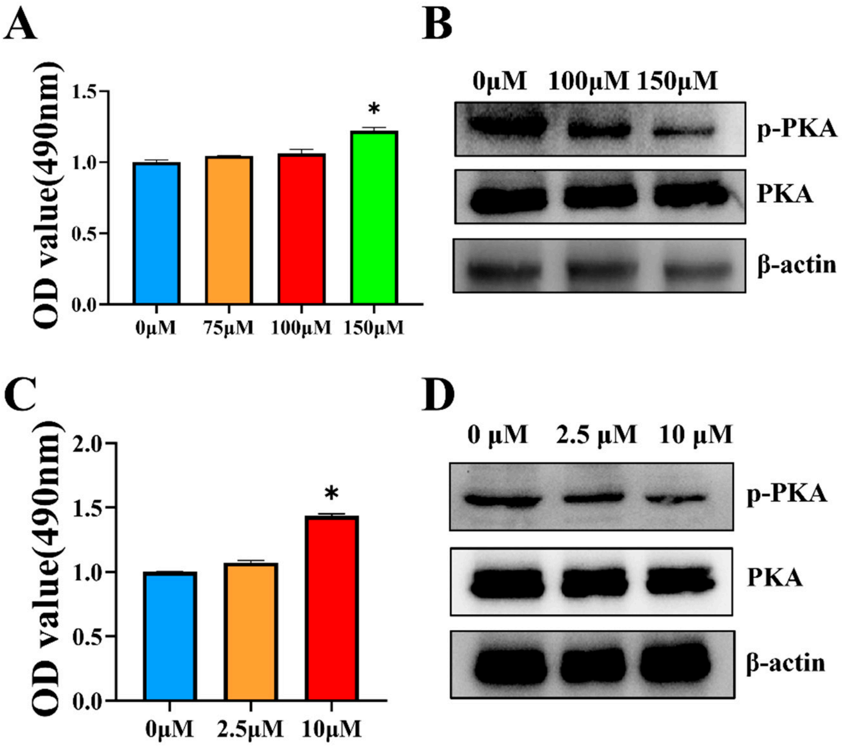

Figure S2. Screening of the optimal effective concentrations of cAMP inhibitor SQ22536 and PKA phosphorylation inhibitor H89 in goat intramuscular preadipocytes. (A) Effects of different concentrations of SQ22536 on cell viability (detected by MTT assay). (B) Effects of different concentrations of SQ22536 on p-PKA and total PKA protein levels (Western blot, β-actin as internal control). (C) Effects of different concentrations of H89 on cell viability (detected by MTT assay). (D) Effects of different concentrations of H89 on p-PKA and PKA protein levels (Western blot, β-actin as internal control). All data are presented as mean ± SEM, $n = 3$. * $p < 0.05$, ** $p < 0.01$.

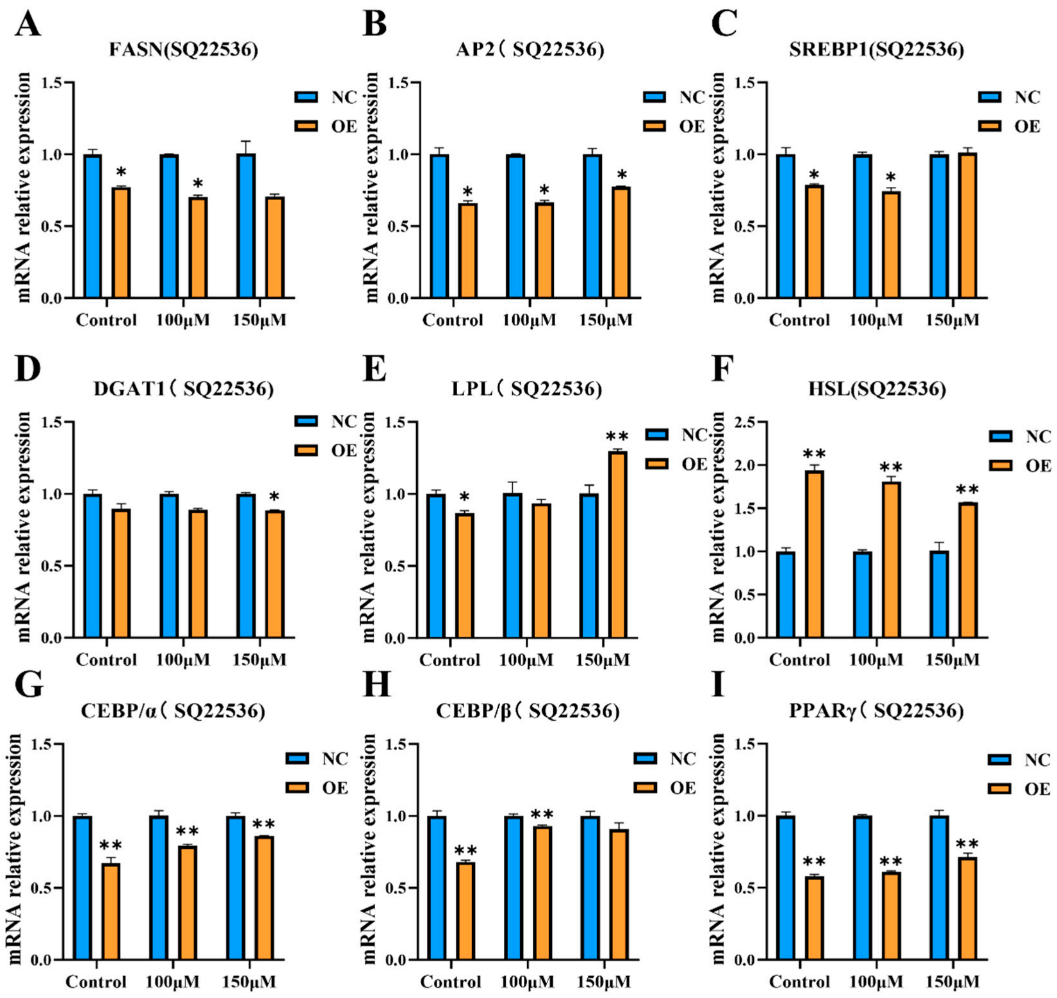

Figure S3. SQ22536 alleviates the inhibitory effect of GPR35 on the adipogenic differentiation of goat intramuscular preadipocytes. (A-I) The mRNA expression levels of adipogenic differentiation marker genes in the NC and OE group, with or without SQ22536 treatment. All data are presented as mean ± SEM, $n = 3$. * $p < 0.05$, ** $p < 0.01$.

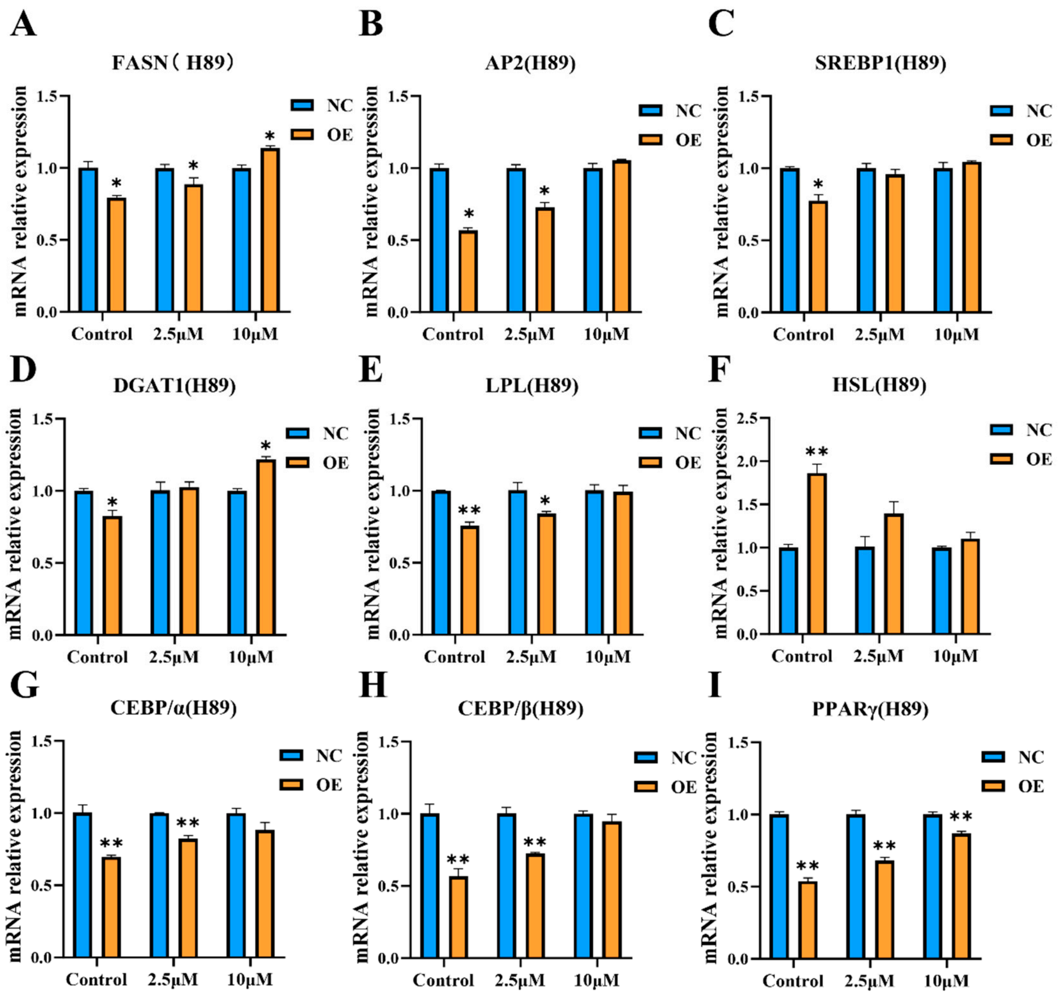

Figure S4. H89 alleviates the inhibitory effect of GPR35 on the adipogenic differentiation of goat intramuscular preadipocytes. (A-I) The mRNA expression levels of adipogenic differentiation marker genes in the NC and OE group, with or without H89 treatment. All data are presented as mean ± SEM, $n = 3$. * $p < 0.05$, ** $p < 0.01$.

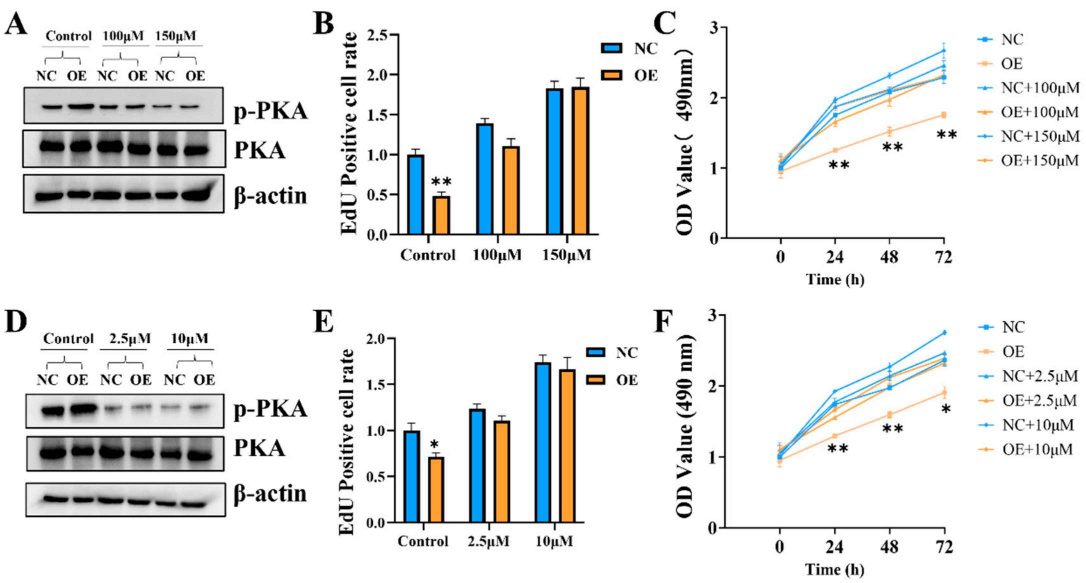

Figure S5. Inhibition of the cAMP/PKA signaling pathway restores GPR35 overexpression-induced proliferation restraint in goat intramuscular preadipocytes. (A) SQ22536 treatment reverses the regulatory effect of GPR35 overexpression on the cAMP/PKA signaling pathway; p-PKA and PKA protein levels detected by Western blot, β-actin as internal control. (B) Semi-quantitative analysis of EDU positive cell counts by ImageJ. (C) Semi-quantitative assessment of cell proliferation via crystal violet staining absorbance detection at 490 nm. (D) H89 treatment reverses the regulatory effect of GPR35 overexpression on the cAMP/PKA signaling pathway; p-PKA and PKA protein levels detected by Western blot, β-actin as internal control. (E) Semi-quantitative analysis of EDU positive cell counts by ImageJ. (F) Semi-quantitative assessment of cell proliferation via crystal violet staining absorbance detection at 490 nm. All data are presented as mean ± SEM, $n = 3$. * $p < 0.05$, ** $p < 0.01$.
